# Chronic muscle stimulation improves muscle function and reverts the abnormal surface EMG pattern in Myotonic Dystrophy: a pilot study

**DOI:** 10.1186/1743-0003-10-94

**Published:** 2013-08-12

**Authors:** Carmelo Chisari, Federica Bertolucci, Stefania Dalise, Bruno Rossi

**Affiliations:** 1 Department of Neuroscience, Unit of Neurorehabilitation, University Hospital of Pisa, Via Paradisa 2, Pisa 56126, Italy

**Keywords:** Myotonic dystrophy type 1, Neuromuscular electrical stimulation (NMES), Rehabilitation, SK = small conductance Ca-activated K + channels

## Abstract

**Background:**

To date, in Myotonic Dystrophy type 1 (DM1) the rehabilitative interventions have always been aimed at muscle strengthening, increasing of fatigue resistance and improving of aerobic metabolism efficiency whereas the electrical membrane fault has always been addressed pharmacologically. Neuromuscular electrical stimulation (NMES) is a useful therapeutic tool in sport medicine and in the rehabilitation of many clinical conditions characterized by motor impairment such as stroke, cerebral palsy and spinal cord injury.

The aim of our pilot study was to evaluate the effects of chronic electrical stimulation both on functional and electrical properties of muscle in a small group of DM1 patients.

**Methods:**

Five DM1 patients and one patient with Congenital Myotonia (CM) performed a home electrical stimulation of the tibialis anterior muscle lasting 15 days with a frequency of two daily sessions of 60 minutes each. Muscle strength was assessed according to the MRC scale (Medical Research Council) and functional tests (10 Meter Walking Test, 6 Minutes Walking Test and Timed Up and Go Test) were performed. We analyzed the average rectified value of sEMG signal amplitude (ARV) to characterize the sarcolemmal excitability.

**Results:**

After the treatment an increase of muscle strength in those DM1 patients with a mild strength deficit was observed. In all subjects an improvement of 10MWT was recorded. Five patients improved their performance in the 6MWT. In TUG test 4 out of 6 patients showed a slight reduction in execution time. All patients reported a subjective improvement when walking. A complete recovery of the normal increasing ARV curve was observed in 4 out of 5 DM1 patients; the CM patient didn’t show modification of the ARV pattern.

**Conclusions:**

NMES determined a clear-cut improvement of both the muscular weakness and the sarcolemmal excitability alteration in our small group of DM1 patients. Therefore this rehabilitative approach, if confirmed by further extensive studies, could be considered early in the management of muscular impairment in these patients. An attractive hypothesis to explain our encouraging result could be represented by a functional inhibition of SK3 channels expressed in muscle of DM1 subjects.

## Background

Myotonia refers to impaired muscle relaxation following a voluntary forceful contraction. It is found in several clinical disorders with different etiologies. Myotonic dystrophy type 1 (DM1) is the most common form of muscular dystrophy in adults affecting approximately 1 in 8000. It is an autosomal dominant inherited disorder with a peculiar and rare pattern of multisystemic clinical features, affecting skeletal muscles, heart, eyes, endocrine and central nervous systems [1].

The genetic basis consist of an expansion of an unstable (CTG)n triplet repetition on chromosome 19 in the 3’ un-translated region (3’-UTR) of the gene encoding for myotonic dystrophy protein kinase (DMPK). The abnormal repeated triplet is situated in a non-coding region of the gene, so it was suggested a loss of function of DMPK protein, caused by either a transcriptional repression or a gain of function mediated by the mutant RNA transcripts. Currently, the best theory explaining the pathogenesis focuses on RNA transcripts: in fact, in DM1 cells were found multiple nuclear foci of mutant DMPK-RNA, containing pathogenic CUG repeats, which could produce defects in alternative splicing of multiple RNAs, thus providing a basis for the multisystemic features of DM1 [2].

The severity of the disease is proportional to the size of the expansion. The number of repetitions tends to increase from generation to generation, accounting for the peculiar genetic anticipation of this disease [3-6].

Another myotonic disorder is Congenital Myotonia (CM), an inherited myotonia due to a mutation in the skeletal muscle chloride channel ClCN1 that leads to reduced sarcolemmal chloride conductance, which in turn allows the muscle to be hyperpolarized, causing delayed relaxation evident as clinical and electrical myotonia [7].

Muscle dysfunction is the most common presenting complaint in DM1. Progressive muscle wasting and weakness are hallmarks of DM1, even if the most characteristic clinical feature of the disease is delayed relaxation of muscle due to repetitive action potentials, a phenomenon called myotonia. Although not the most serious complication of DM1, myotonia exacerbates disability, with preferential involvement of hands and forearms [8].

There is no cure for DM1. Current treatment for DM1 is limited to supportive care that partially alleviates signs and symptoms of the disease but does nothing to slow or halt disease progression. To date, mexiletine, which modulates sodium channels and thus lessens myotonia, and Central Nervous System stimulants to address fatigue, are the only useful drugs routinely applied in practice [9]. At once, research in the field has led to significant advances in understanding the complex pathophysiology of the disease. Consequently, numerous approaches to targeting disease mechanisms at each of the steps in the pathogenesis at RNA level are demonstrating great promise in pre-clinical studies [10].

In the last 10 years increasing attention has been paid on rehabilitation in Neuromuscular Disorders but unfortunately the data are not so strong to provide clear guidelines [11].

The phenotypic expression of myotonia and dystrophy is variously combined in patients and the pathophysiological mechanism underlying the muscle impairment remains unclear. Different pathophysiological hypothesis have been formulated in this regard; overall, the impairment is thought to be due to an alteration of the transmembrane ionic currents. Currently, the most shared hypothesis attributes the hyperexcitability of skeletal muscle to a reduced conductance of chloride channels that leads to a sarcolemmal excitability alteration [12]. Using skeletal muscle from a transgenic mouse model of DM1, Mankodi and colleagues [12] showed that expression of expanded CUG repeats in skeletal muscle reduces the transmembrane chloride conductance to levels consistent with those expected to cause myotonia. Additional studies determined that aberrant splicing of the chloride channel ClCN1 resulted in loss of ClCN1 protein from the surface membrane [13]. Charlet and colleagues [14] demonstrated that CUG-Binding Protein (CUG-BP), which is elevated in DM1 skeletal muscle, binds to the ClCN1 pre-mRNA, and overexpression of CUG-BP in transfected cells reproduces the aberrant pattern of ClCN1 splicing. These groups propose that disruptions in alternative splicing regulation of ClCN1 causes a channelopathy and membrane hyperexcitability, leading to the classic DM1 feature of myotonia [12,14].

However, a very attractive theory is based on the abnormal presence of small conductance Ca-activated apamin-sensitive K + channels (SK3) in DM1 muscle [15]. Calcium-activated potassium channels are an heterogeneous family widely distributed in neurones and peripheral tissues, controlling repetitive firing in excitable cells and secretion in exo- and endocrine cells [16,17]. A subset of this family are the small conductance ones (SK), first recorded at the single channel level by Blatz & Magleby [18], voltage independent and found to underlie the long lasting after-hyperpolarization (AHP) following the action potential [19]. SK channels are comprised of several types (SK1, SK2, SK3) encoded by specific genes. SK3 channels are abnormally expressed in DM1 muscle but not in CM muscle [20]. They have been also revealed in myotubes and in adult denervated muscle [21]. Interestingly, Beherens and Vergara (1994) [22] showed that injection of apamin, a bee venom toxin peptide which is a highly selective ligand and functional blocker of SK channels, in the thenar eminence muscles reduces EMG myotonic discharges in DM1. A similar effect has been demonstrated in our previous studies in which we showed that the local application of apamin produces functional modifications in DM1 muscle both on needle EMG “myotonic runs” and on the characteristic surface EMG pattern [23,24].

Very recently an interesting study in rats showed that chronic Neuromuscular Electrical Stimulation (NMES) of denervated soleus muscles determines down-regulation of SK3 channels which are known to be expressed by adult muscle after denervation [21,25].

NMES is a useful therapeutic tool in sport medicine and in the rehabilitation of many clinical conditions characterized by motor impairment [26,27] such as stroke, cerebral palsy and spinal cord injury [28-30].

Starting from these assumptions, the aim of our study was to evaluate the effects of a chronic electrical stimulation protocol in a small group of DM1 patients both on functional and electrical properties of muscle.

## Methods

Five DM1 patients (3 males and 2 females) and one patient with the dominant autosomal form of Congenital Myotonia (CM) were enrolled (Table 1). The age range was 28-65 years. Molecular DNA analysis for CTG triplet repetitions on chromosome 19 was performed in all DM1 patients. The main clinical features of the patients are summarized in Table 1. All subjects gave their informed consent prior to testing and the study was approved by the Ethical Committee of University Hospital of Pisa.

**Table 1:** Patient description and functional results

			**MRC**		**10MWT (min)**		**6 MWT (m)**		**TUG (min)**		**F index values**	
**Patient**	**Sex**	**Age (years)**	**T0**	**T1**	**T0**	**T1**	**T0**	**T1**	**T0**	**T1**	**T0**	**T1**
1 DM1	M	35	5	5	7,54	5,34	364	402	8,78	8,75	0,59	-0,11
2 DM1	F	28	4	5	8,18	6,87	394	353	8,92	8,92	0,23	-0,06
3 DM1	M	65	4	5	8,98	6,25	411	465	8,1	8,22	0,3	-0,33
4 DM1	M	28	4	5	11,89	11,55	322	340	12,6	12,12	0,31	0,07
5 DM1	F	58	3	3	15,3	14,44	285	301	13,67	13,56	-0,23	-0,27
6 CM	M	39	5	5	8,43	7,88	438	475	8,76	8,5	0,65	0,58

Patients description, functional assessment and F Index before (T0) and after 15 days (T1) of electrical stimulation protocol. *DM1* Myotonic Distrophy type 1, *CM* Congenital Myotonia, *MRC* Medical Research Council, *10MWT* 10 Meters Walking Test, *6MWT* 6 Minutes walking Test, *TUG* Timed Up and Go test, *min* minutes, *m* metres.

### Functional assessment

Patients were assessed for muscle strength of tibialis anterior (TA) according to the MRC scale (Medical Research Council). Motor function was explored through the 10 Meter Walking Test (10MWT), the 6 Minutes Walking Test (6MWT) and the Timed Up&Go Test (TUG).

### Surface EMG assessment

#### Experimental procedure

Each subject was made to lie on a bed with one knee fully extended and the relative ankle joint at 110°. The motor points of the muscle were indentified as those with the lowest stimulation threshold. The number of motor points ranged from one to three: the most distal one was used for the stimulation protocol.

The detection electrode was applied to previously shaved skin cleansed with alchol. No conduction paste was necessary. The electrode was moved over the muscle in the area between the most distal motor point and the tendon and was positioned, with an elastic strap, with the two bar approximately perpendicular to the Tibialis Anterior belly direction. A sensor for skin temperature with a resolution of 0,1°C was fixed on the skin, near the detection electrode, to check that skin temperature did not change more than ±0,5°C during the experiment. A pause of about 5 min was allowed before beginning the experimental phase in order to avoid any fatigue effect.

Stimulated contractions were then performed with the subjects relaxed and physically passive; the absence of voluntary ME signals indicated this state.

#### Motor point stimulation

We used a motor point stimulation protocol to characterize sarcolemmal excitability according to previous study of Chisari et al. [23,24].

Briefly, the stimulation was applied using a monopolar technique with a negative, soaked-sponge electrode (2 × 3 cm2) placed on the motor point and a large (8 × 12 cm2) positive electrode on the gastrocnemius muscle [31]. The frequency of stimulation was 35 Hz, which is the highest one that records sequentially muscle potential action without M-waves overlapping. A supramaximal stimulation, 10-15% above the level generating the maximal amplitude of motor evoked potential, measured through surface EMG M wave, was then applied.

#### EMG recording

The myoelectric signal was detected by means of 2-bar electrode. These bars, 10 mm long and 2 mm thick, are 10 mm apart and are fixed on a mildly flexible support. A single differential output was obtained from the two bars and was used to compute the myoelectric signal, which was amplified, low-pass filtered with a cut-off frequency of 480 Hz, digitized by a 12 bit analog-digital converter and stored on the disk of a PC. The signal was then tabulated and correlated with time for each stimulated contraction.

We analyzed the average rectified value of EMG signal amplitude (ARV) expressed in microvolts. We utilized the DEM (Dispositivi Elettro Medicali – Torino – Italy) Surface Myoelectric Signals Stimulation and Detection System.

### NMES protocol

The patients performed a home electrical stimulation of the tibialis anterior muscle through a train of biphasic rectangular pulses, duration of 0.1 msec, 20 Hz frequency and supramaximal intensity. A bipolar stimulation technique was used, with electrodes placed along course of muscle (negative electrode to proximal and positive electrode to distal level) and fixed with self-adhesive strip. The stimulation was intermittent, consisting of an active phase of contraction (phase on) lasting 10 sec, followed by a 10 sec resting phase (phase off).

Treatment program lasted 15 days, with a frequency of two daily sessions of 60 minutes each. The evaluations were performed at T0 (before training) and T1 (after 15 days of training). Two patients (1 and 2) continued the stimulation till 30 days (T2) In these patients the sEMG signals were also recorded 15 days after suspension (T3).

### Data analysis

Quantitative evaluation of the ARV pattern was performed through computation of the F index (or area ratio index), a regression-free muscle fatigue index based on the rate of change of measured surface EMG variables described by Merletti et al. [32]. F index is the product of a reference value (e.g., the first value of the time series) and the time of observation defines a reference rectangle. The area between the upper side of such rectangle and the experimental data points is divided by the area of the reference rectangle to provide this index. It is regression-free, it is dimensionless, it varies between 0 and 1 for decreasing patterns and it is negative for increasing patterns [32].

As previously reported by Chisari et al. [23] the surface EMG amplitude parameter (ARV) exhibits an increasing trend in normal subjects but a decreasing one in DM1 patients, during prolonged contraction. Accordingly, the F index assumes negative or positive values in normal or pathological trends, respectively. The change in the F index from positive to negative values was taken as a criterion of the efficacy of the treatment.

## Results

After treatment patients 2, 3 and 4 showed an increase in muscle strength at T1 (MRC 4 to MRC 5); no change was observed in other patients. In all subjects an improvement of 10MWT was recorded. In TUG test 4 out of 6 patients (patients 1, 4, 5 and 6) showed a slight reduction in execution time. Five patients, (patients 1, 3, 4, 5, 6) improved their performance in the 6MWT (Table 1). All patients reported a subjective improvement when walking.

**Figure 1:**
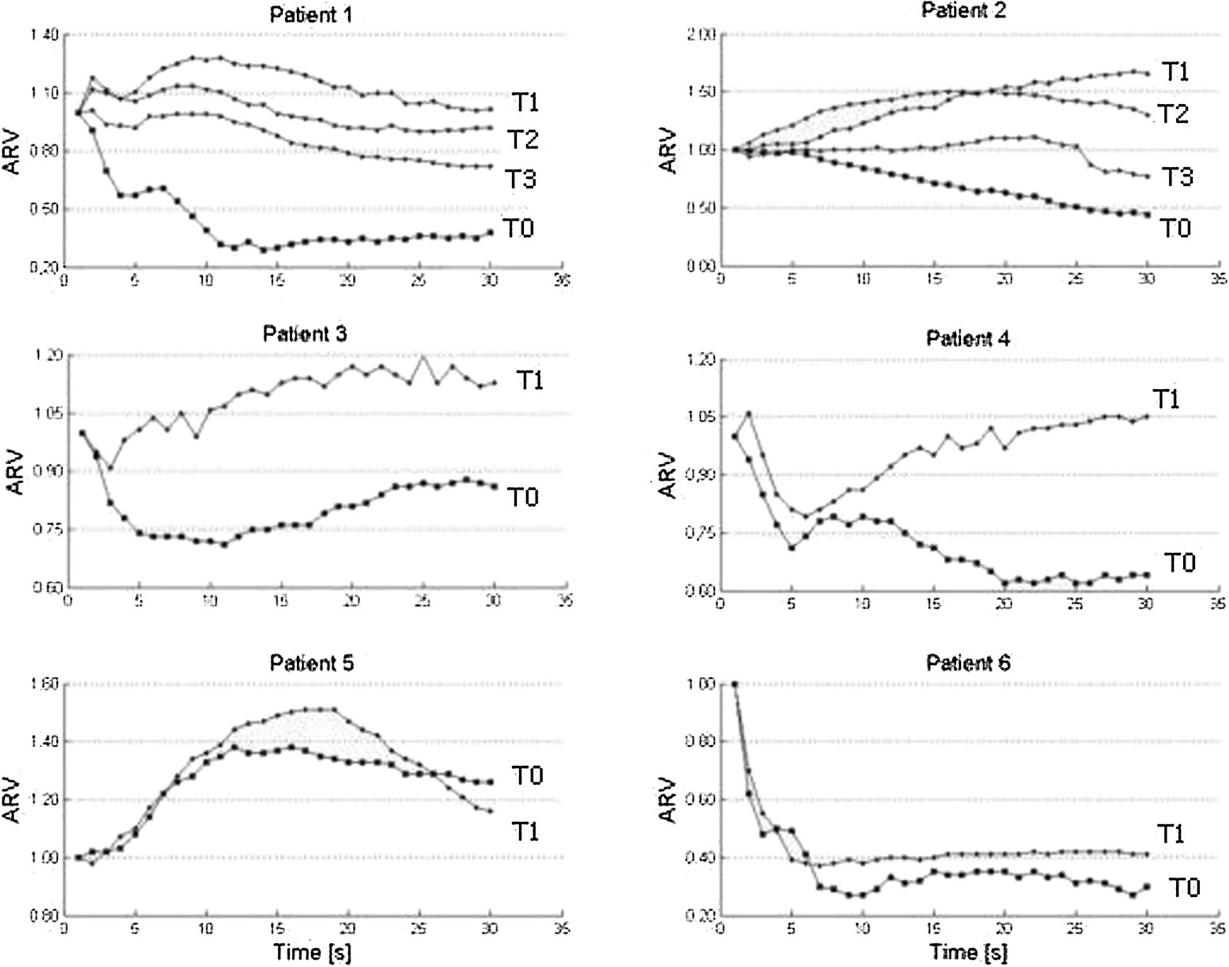
**Normalized ARV values before and after the treatment.** ARV = Average Rectified Value of EMG signal amplitude. On the *x axis* is represented Time (in seconds); on *y axis* is represented the ARV value. In the figure legend, T0 = before training; T1 = after 15 days of training; T2 = after 30 days of training; T3 = 15 days after suspension.

As regard the EMG assessment (Figure 1), patients 1, 2, 3, 4 and 6 showed the typical decreasing ARV pattern at T0 whereas patient 5 exhibited an increasing trend (as expected because of his severe strength deficit). A clear and complete recovery of the normal increasing ARV curve was observed at T1 in pts 1, 2, 3 and 4, with F index changing from positive to negative value. In patients 1 and 2 a normal ARV trend, with a negative F index, could still be recorded at T2. Recording of ARV at T3 (15 days after the end of stimulation) showed a trend toward a pathological pattern with the F index coming back to a positive value. Patients 5 and 6 did not show modification of the ARV pattern at T1, data confirmed by an unchanged F Index (Table 1).

## Discussion

The results of this pilot study show that neuromuscular electrical stimulation (NMES) in patients affected by Myotonic Dystrophy type 1 and Congenital Myotonia determines a clear improvement of muscular impairment.

The improvement of muscle strength observed after the treatment in those DM1 patients with a mild strength deficit is a very important clinical datum per se. Indeed, they present a high risk of falls because of weakness in specific muscle groups (for example knee extensors and mostly ankle/toe dorsi-flexors) [33]; therefore, an early intervention through NMES could be a fundamental approach in order to reduce this risk.

On the other hand we observed after the treatment an improvement in the 10MWT, meaning an increase in walking velocity, in all patients. 5 out 6 patients showed a better performance in the 6MWT.

Finally in 4 pts we recorded a reduction in time to complete the Timed Up and Go Test. This test involves a higher number of muscle group and this could explain the mild improvement observed only in 4 out 6 patients. We have to keep in mind that we stimulated the Tibialis Anterior muscle that is a very important muscle for walking but is not so influent to stand up from a chair.

These functional findings, though obtained in a limited number of subjects and showing a transient effect, seem very interesting because of their possible effect on the Quality of Life of DM1 and CM patients.

An effect on sarcolemmal excitability of DM1 patients is demonstrated by the sEMG assessment which showed, after the NMES protocol, a clear and complete recovery of the normal increasing ARV curve at T1 in patients 1, 2, 3, 4. A normal ARV trend could still be recorded at T2 in patients 1 and 2. Patient 5 (the patient with the most severe strength deficit, as shown by MRC), as expected because of her increasing trend at T0, and CM subject (pt 6) didn’t show modification of the ARV pattern. In previous studies Chisari et al. [24] found a clear-cut relationship between the ARV trend and the sarcolemmal excitability alteration and/or the myofibre degeneration contributing to muscle impairment in DM1. In particular, a decreasing ARV trend (typical of patient without strength deficit or with a mild one) is closely related to the sarcolemmal excitability alteration whereas a normal increasing trend, observed in those patients with severe strength deficit, refers to a reduced number of myofiber and/or to the atrophic process, characteristic of DM1 muscle in the later phase of the disease. Therefore the rescue of a normal EMG pattern in DM1 patients 1, 2, 3, 4 and the lack of effect in patient 5 indicates the specificity of the effect of NMES on the membrane excitability. Moreover the EMG pattern of CM patient did not show any change after the stimulation. This suggests an effect on a membrane excitability defect which is peculiar of DM1.

NMES protocols consist of a combination of pulse parameters and time modulations variously combined in order to induce muscle contraction that aim to stimulate both endurance and strength training [26,27]. The selection of stimulation parameters is typically based on each patient’s rehabilitation goal. For example, stimulation frequencies <15 Hz help increasing aerobic capacity in patients with heart failure while stimulation frequencies >50 Hz are able to increase muscle strength [31]. The mechanisms underlying these effects have been studied extensively and they seem to be due to different processes as an increase of cross-sectional area of the stimulated muscle [31], an activation of additional motor units [34] and a metabolic activation of muscle fibres through an increase in the number of cross-bridges between actin and myosin myofilaments [35].

Giving an exhaustive explanation of the operating mechanism by which NMES acted in our patients is difficult, as we adopted an unusual frequency stimulation and as we do not have histological and/or molecular data of the treated muscles. A cooperation of all the factors mentioned above could have occurred. Nevertheless, another attractive hypothesis comes out from the parallelism with a recent study on a denervated muscle animal model [25] which showed that chronic stimulation in vivo is able to revert the denervation-induced up-regulation of SK3 channels expression. In that experiment chronic electrical stimulation of denervated muscles also completely prevented the development of the after-hyperpolarization (AHP) following the action potential, normally induced in the muscle fibers by denervation.

On this background an interesting speculation to explain our encouraging result obtained in DM1 patients could be represented by a functional inhibition and/or a down regulation, by means of NMES, of SK3 channels, abnormally expressed in muscle of these patients. This hypothesis is reinforced by the evidence of inefficacy of the treatment on the EMG pattern recorded in CM patient; in fact in CM muscles is not documented the abnormal expression of SK3 channels [20].

Of course further studies are necessary in order to verify this suggestive hypothesis. Future tests should take into account the study of Rhodes et al. [36] describing the effect of apamin, a highly selective ligand and functional blocker of SK channels, on DM1 lens cell lines [36]. These authors found an increased resistance in subsequent culture transfers and therefore a higher survival in cell lines incubated with apamin. They concluded that apamin protects the cell lens from degeneration. Moreover they suggested that also atrophy and degeneration of muscle tissue could be due to SK3 channels expression. Considering these data of Rhodes et al. [36], the availability of a rehabilitative tool able to interfere with the expression of SK3 channels could have a possible preventive action on the “dystrophic” evolution of the disease.

## Conclusions

In conclusion, the results of our pilot study show that Neuromuscular Electric Stimulation in patients affected by Myotonic Dystrophy type 1 determines an improvement of both the muscular weakness and the sarcolemmal excitability alteration. This new therapeutic approach could be able to reduce the risk of falls in these patients. It can be comfortably performed at home, therefore could be considered early in the management of muscular impairment in DM1. Of course we cannot disregard that we examined a small group of patients and a largest cohort of patients have to be investigated to confirm these data. Moreover no molecular studies have been performed in order to clarify the intrinsic effect of NMES. But, if our pathogenetic hypothesis were confirmed, it would be desirable to intensify the research about a feasible preventive role of this rehabilitative method on DM1 progression.

## Abbreviations

DM1: Myotonic Dystrophy type 1; 3’-UTR: 3’ un-translated region; DMPK: Myotonic Dystrophy protein kinase; CM: Congenital Myotonia; ClCN1: Chloride Channel 1; CUG-BP: CUG-Binding Protein; SK: Ca-activated apamin-sensitive K + channels; EMG: Electromyography; ARV: Average Rectified Value; NMES: Neuromuscular Electrical Stimulation; VM: Vastus Medialis; TA: Tibialis Anterior; MRC: Medical Research Council; 10MWT: 10 Meter Walking Test; 6MWT: 6 Minutes Walking Test; TUG: Timed Up&Go Test.

## Competing interests

No competing interests exist.

## Authors’ contributions

Each author participated sufficiently in the work to take public responsibility for the content. All authors read and approved the final manuscript.

## References

[B1] Shaw DJ Harper PS Myotonic dystrophy: developments in molecular genetics Br Med Bull 1989 45 3 745 759 2688826 10.1093/oxfordjournals.bmb.a072355

[B2] Cho DH Tapscott SJ Myotonic Dystrophy: Emerging mechanisms for DM1 and DM2 Biochim Byophisica Acta 2007 1772 195 204 10.1016/j.bbadis.2006.05.013 16876389

[B3] Brook JD McCurrach ME Harley HG Molecular basis of myotonic dystrophy: expansion of a trinucleotide (CTG) repeat at the 3′ end of a transcript encoding a protein kinase family member Cell 1992 68 799 808 10.1016/0092-8674(92)90154-5 1310900

[B4] Fu YH Pizzuti A Fenwick RG Jr An unstable triplet repeat in a gene related to myotonic muscular dystrophy Science 1992 255 5049 1256 1258 10.1126/science.1546326 1546326

[B5] Mahadevan M Tsilfidis C Sabourin L Myotonic dystrophy mutation: an unstable CTG repeat in the 3' untranslated region of the gene Science 1992 255 5049 1253 1255 10.1126/science.1546325 1546325

[B6] Aslanidis C Jansen G Amemiya C Cloning of the essential myotonic dystrophy region and mapping of the putative defect Nature 1992 355 6360 548 551 10.1038/355548a0 1346925

[B7] Crews J Kaiser KK Brooke MH Muscle pathology of myotonia congenita J Neurol Sci 1976 28 4 449 457 10.1016/0022-510X(76)90116-7 133210

[B8] Thurman MW Myotonic Dystrophy: Therapeutic strategies for the future Neurotherapeutics 2008 5 592 600 10.1016/j.nurt.2008.08.001 19019311 PMC4514697

[B9] Logigian EL Martens WB Moxley RT McDermott MP Dilek N Wiegner AW Pearson AT Barbieri CA Annis CL Thornton CA Moxley RT 3rd Mexiletine is an effective antimyotonia treatment in myotonic dystrophy type 1 Neurology 2010 74 18 1441 1448 10.1212/WNL.0b013e3181dc1a3a 20439846 PMC2871004

[B10] Pennock Foff E Mahadevan MS Therapeutics Development in Myotonic Dystrophy Type I Muscle Nerve 2011 44 2 160 169 10.1002/mus.22090 21607985 PMC3136655

[B11] Cup EH Pieterse AJ Ten Broek-Pastoor JM Munneke M van Engelen BG Hendricks HT van der Wilt GJ Oostendorp RA Exercise therapy and other types of physical therapy for patients with neuromuscular diseases: a systematic review Arch Phys Med Rehabil 2007 88 11 1452 1464 10.1016/j.apmr.2007.07.024 17964887

[B12] Mankodi A Takahashi MP Jiang H Beck CL Bowers WJ Moxley RT Cannon SC Thornton CA Expanded CUG repeats trigger aberrant splicing of ClC-1 chloride channel pre-mRNA and hyperexcitability of skeletal muscle in myotonic dystrophy Mol Cell 2002 10 1 35 44 10.1016/S1097-2765(02)00563-4 12150905

[B13] Ebralidze A Wang Y Petkova V Ebralidse K Junghans RP RNA leaching of transcription factors disrupts transcription in myotonic dystrophy Science 2004 303 383 387 10.1126/science.1088679 14657503

[B14] Charlet BN Savkur RS Singh G Philips AV Grice EA Cooper TA Loss of the muscle-specific chloride channel in type 1 myotonic dystrophy due to misregulated alternative splicing Mol Cell 2002 10 45 53 10.1016/S1097-2765(02)00572-5 12150906

[B15] Renaud JF Desnuelle C Schmid-Antomarchi H Hugues M Serratrice G Lazdunski M Expression of apamin receptor in muscles of patients with myotonic muscular dystrophy Nature 1986 319 6055 678 680 10.1038/319678a0 2419758

[B16] Blatz AL Magleby KL Calcium-activated potassium channels Trends Neurosci 1987 10 463 467 10.1016/0166-2236(87)90101-9

[B17] Vergara C Latorre R Marrion NV Adelman JP Calcium-activated potassium channels Curr Opin Neurobiol 1998 8 321 329 10.1016/S0959-4388(98)80056-1 9687354

[B18] Blatz AL Magleby KL Single apamin-blocked Ca-activated K + channels of small conductance in cultured rat skeletal muscle Nature 1986 323 718 720 10.1038/323718a0 2430185

[B19] Sah P Ca2 + -activated K + currents in neurons: types, physiological roles and modulation Trends Neurosci 1996 19 150 154 10.1016/S0166-2236(96)80026-9 8658599

[B20] Cleland JC Griggs R Treatment of Neuromuscular Channelopathies: Current Concepts and Future Prospects Neurotherapeutics 2008 5 607 612 10.1016/j.nurt.2008.09.001 19019313 PMC4514704

[B21] Pribnow D Johnson-Pais T Bond CT Keen J Johnson RA Janowsky A Silvia C Thayer M Maylie J Adelman JP Skeletal muscle and small-conductance calcium-activated potassium channels Muscle Nerve 1999 22 6 742 750 10.1002/(SICI)1097-4598(199906)22:6<742::AID-MUS11>3.0.CO;2-1 10366228

[B22] Behrens MI Jalil P Serani A Vergara F Alvarez O Possible role of apamin-sensitive K + channels in myotonic dystrophy Muscle Nerve 1994 17 11 1264 1270 10.1002/mus.880171104 7935548

[B23] Chisari C Licitra R Pellegrini M Pellegrino M Rossi B Fluoxetine blocks myotonic runs and reverts abnormal surface electromyogram pattern in patients with myotonic dystrophy type 1 Clin Neuropharmacol 2009 32 6 330 334 10.1097/WNF.0b013e3181ae5546 19667977

[B24] Chisari C Simonella C Rossi B A surface EMG analysis of sarcolemma excitability alteration and myofibre degeneration in Steinert disease Clin Neurophysiol 2001 112 10 1925 1930 10.1016/S1388-2457(01)00619-8 11595153

[B25] Favero M Jiang DJ Chiamulera C Cangiano A Fumagalli GF Expression of small-conductance calcium-activated potassium channels (SK3) in skeletal muscle: regulation by muscle activity J Physiol 2008 586 19 4763 4774 10.1113/jphysiol.2008.156588 18703580 PMC2614046

[B26] Delitto A Brown M Strube MJ Rose SJ Lehman RC Electrical stimulation of quadriceps femoris in an elite weight lifter: a single subject experiment Int J Sports Med 1989 10 187 191 10.1055/s-2007-1024898 2674035

[B27] Dudley GA Castro MJ Rogers S Apple DF Jr A simple means of increasing muscle size after spinal cord injury: a pilot study Eur J Appl Physiol Occup Physiol 1999 80 394 396 10.1007/s004210050609 10483812

[B28] Maffiuletti NA Cometti G Amiridis IG Martin A Pousson M Chatard JC The effects of electromyostimulation training and basketball practice on muscle strength and jumping ability Int J Sports Med 2000 21 437 443 10.1055/s-2000-3837 10961520

[B29] Snyder-Mackler L Delitto A Stralka SW Bailey SL Use of electrical stimulation to enhance recovery of quadriceps femoris muscle force production in patients following anterior cruciate ligament reconstruction Phys Ther 1994 74 901 907 8090841 10.1093/ptj/74.10.901

[B30] Stackhouse SK Binder-Macleod SA Stackhouse CA McCarthy JJ Prosser LA Lee SC Neuro-muscular electrical stimulation versus volitional isometric strength training in children with spastic diplegic cerebral palsy: a preliminary study Neurorehabil Neural Repair 2007 21 6 475 485 10.1177/1545968306298932 17369515 PMC3069852

[B31] Bax L Staes F Verhagen A Does neuromuscular electrical stimulation strengthen the quadriceps femoris? A systematic review of randomised controlled trials Sports Med 2005 35 191 212 10.2165/00007256-200535030-00002 15730336

[B32] Merletti R Lo Conte LR Orizio C Indices of muscle fatigue J Electromyogr Kinesiol 1991 1 1 20 33 10.1016/1050-6411(91)90023-X 20719592

[B33] Wiles CM Falls and stumbles in myotonic dystrophy J Neurosurg Psychiatr 2006 77 393 396 10.1136/jnnp.2005.066258 PMC2077718 16199443

[B34] Binder-Macleod SA Snyder-Mackler L Muscle fatigue: clinical implications for fatigue assessment and neuromuscular electrical stimulation Phys Ther 1993 73 902 910 8248298 10.1093/ptj/73.12.902

[B35] Deley G Kervio G Verges B Comparison of low-frequency electrical myostimulation and conventional aerobic exercise training in patients with chronic heart failure Eur J Cardiovasc Prev Rehabil 2005 12 226 233 15942420 10.1097/01.hjr.0000166455.23346.a5

[B36] Rhodes JD Nobckton DG McAbney JP Prescott AR Duncan G Increased SK3 expression in DM1 lens cells leads to impaired growth throught a greater calcium-induced fragility Hum Mol Genet 2006 15 3559 3568 10.1093/hmg/ddl432 17101631

